# Pneumonectomy for Unilateral Proximal Interruption of Pulmonary Artery: A Case Series from the Literature

**DOI:** 10.3390/life13122328

**Published:** 2023-12-12

**Authors:** Giovanni Mattioni, Mario Nosotti, Lorenzo Rosso, Alessandro Palleschi, Davide Tosi, Paolo Mendogni

**Affiliations:** 1 Thoracic Surgery and Lung Transplantation Unit, IRCCS Foundation Ca’ Granda Ospedale Maggiore Policlinico, 20122 Milan, Italy; mario.nosotti@unimi.it (M.N.); lorenzo.rosso@unimi.it (L.R.); alessandro.palleschi@unimi.it (A.P.); davide.tosi@policlinico.mi.it (D.T.); paolo.mendogni@policlinico.mi.it (P.M.); 2 School of Thoracic Surgery, University of Milan, 20122 Milan, Italy

**Keywords:** unilateral proximal interruption of pulmonary artery (UPIPA), pulmonary artery agenesis, pneumonectomy, congenital, vascular malformations

## Abstract

Background: Unilateral proximal interruption of the pulmonary artery (UPIPA) is a rare congenital disease, and its optimal management remains undefined in the existing literature. The occasional necessity for pneumonectomy is still supported by limited evidence. Methods: A systematic review of the literature was conducted using the PubMed search engine, focusing on UPIPA cases that received pneumonectomy. Thirty-one pertinent articles were selected and included in the analysis. A case reported from our institution was included in the analysis. Results: We found 25 adults and seven children affected by UPIPA who received an indication for pneumonectomy, plus an additional case that was reported by our institution. Among adult patients, the predominant indication was hemoptysis (57%), followed by suspected or confirmed lung cancer (23%). Approximately 46% of surgical procedures were classified as urgent or emergent. Postoperative complications were observed in 36% of cases, with no recorded mortality. In pediatric cases, pneumonectomy was primarily a life-saving intervention, performed urgently or emergently in 75% of instances. A possible late complication in pediatric patients involves a mediastinal shift leading to respiratory distress, which may be mitigated using an inflatable prosthesis. Conclusions: Pneumonectomy achieves complete resolution of UPIPA symptoms. In the adult population, its primary indication is hemoptysis, with procedures conducted in both elective and urgent/emergent settings. Despite a mortality rate of zero, a notable proportion of patients may experience postoperative complications. In pediatric cases, the clinical presentation varies more extensively, and pneumonectomy is typically reserved for life-threatening situations, emphasizing the need for careful patient selection.

## 1. Introduction

Unilateral absence of pulmonary artery (UAPA), more precisely denoted as unilateral proximal interruption of pulmonary artery (UPIPA) or ductal origin of pulmonary artery (DOPA), represents a rare congenital anomaly characterized by the absence of the proximal segment of the main pulmonary artery (PA) on one side. The distal segment may originate from a ductus arteriosus arising from a systemic circulation artery, most frequently the brachiocephalic artery and the left subclavian artery [1]. The patency of this connection exhibits variability, with the likelihood of its persistence decreasing as age advances. UPIPA may be isolated or associated with various cardiovascular congenital anomalies. The clinical presentation displays considerable variability, and, currently, optimal management has not been established. Management may drastically change depending on the characteristics of the disorder and the patient. While pneumonectomy has been sporadically undertaken in UPIPA patients [2,3], its role as a curative treatment for UPIPA remains uncertain. To the best of our knowledge, there are no studies in the literature addressing this topic. In this article, we present a systematic review of pneumonectomies performed in patients affected by UPIPA, encompassing both adult and pediatric populations. Our objective is to aggregate evidence pertaining to the indications, outcomes, and follow-up considerations for this procedure. This article is presented in accordance with the PRISMA reporting checklist [4].

## 2. Materials and Methods

**Figure 1:**
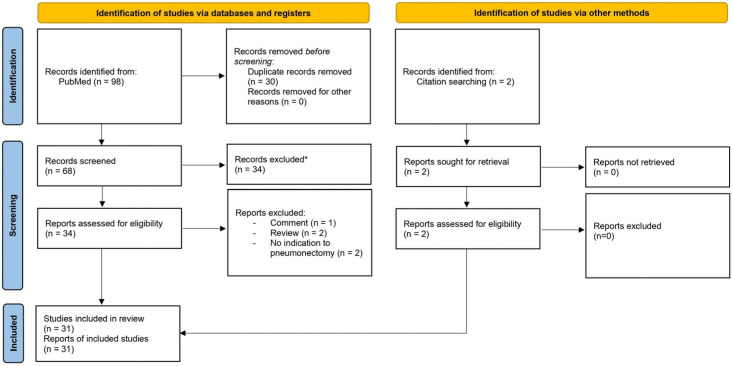
PRISMA 2020 flow diagram for new systematic reviews, which included searches of databases, registers, and other sources. For more information, visit http://www.prisma-statement.org/ (accessed on 9 March 2023). * Non-inherent to unilateral proximal interruption of pulmonary artery.

A systematic literature search was conducted using the PubMed search engine that included articles published between 1 January 1990 and 1 October 2023. The search strings used were [“unilateral” AND “pulmonary arter*” AND (agenesis OR absen* OR “proximal interruption” OR atresia) AND “pneumonectomy”] = 59 results, [“pulmonary arter*”[ti] AND (agenesis[ti] OR absen*[ti] OR “proximal interruption”[ti] OR atresia[ti]) AND “pneumonectomy”] = 39 results, and [“pulmonary” AND (arter*) AND “ductal origin” AND “pneumonectomy”] = 0 results. We included papers concerning patients affected by unilateral proximal interruption of pulmonary artery that received an indication for and/or underwent pneumonectomy. Only articles in English were selected. The PRISMA flow diagram of the study selection is shown in Figure 1 [4]. The preliminary search of titles and abstracts yielded 98 papers, of which 64 were removed for the reasons reported in the flow diagram. The remaining articles were selected according to the inclusion and exclusion criteria [5,6,7,8,9,10,11,12,13,14,15,16,17,18,19,20,21,22,23,24,25,26,27,28,29,30,31,32,33]. Two additional articles were found in the references of reviewed articles [34,35]; as a result, 31 articles were considered. When follow-up data were not available or not recent, the corresponding author was contacted via e-mail. Additional data were therefore obtained from one study [17]. A case reported from our institution was included in the analysis (see details in Supplementary Materials). The Rayyan application was used to elaborate the present review [36].

In this paper, we use the definition of unilateral proximal interruption of pulmonary artery (UPIPA) because we believe this description easily encompasses the main clinical and surgical issues of this disease.

## 3. Results

From the 31 selected articles, we found a total of 32 patients who required a right or left pneumonectomy for UPIPA, of whom 25 were adults and 7 were children. Articles were case reports and case series.

### 3.1. Pneumonectomy in Adult Population

In the current literature, 25 adult patients received an indication for pneumonectomy for UPIPA. One patient had his operation aborted intraoperatively, and an additional case from our institution is included in this report. Finally, a total of 25 patients underwent pneumonectomy. Patients’ characteristics are reported in Table 1 and Supplementary Table S1. The mean age at the time of the surgery was 39.2 years (median 35 years, range 19–70 years). Fifteen (58%) were males. There were 15 (58%) right UPIPA and 11 (42%) left UPIPA. Associated cardiovascular congenital anomalies were present in nine (35%) patients. Of them, eight (89%) had a right aortic arch (all with left UPIPA), and one had an aberrant subclavian artery with Kommerell’s diverticulum. In addition, one patient exhibited a single left pulmonary vein. Among the 26 patients at the time of pneumonectomy indication, only 1 (4%) was asymptomatic. The indications for pneumonectomy were:

- Massive acute hemoptysis in seven (27%) cases, with two occurring post-failed percutaneous embolization and three after percutaneous embolization was either aborted or deemed not possible. Two cases also presented with respiratory failure;
- Suspected or diagnosed tumor in six (23%) cases;
- New-onset of acute hemoptysis in four (15%) cases, one following a failed percutaneous embolization;
- Persistent/recurrent hemoptysis in four (15%) cases;
- A combination of hemoptysis, recurrent respiratory tract infections, and dyspnea in three (12%) cases;
- Recurrent respiratory infections in the setting of a damaged lung in one (4%) case;
- Myocardial ischemia for coronary steal in one (4%) case.

Surgery was elective in 14 (54%) patients and urgent/emergent in 12 (46%). In three (12%) cases, pneumonectomy was performed as a life-saving procedure. One pneumonectomy was aborted intraoperatively due to the discovery of metastatic lung cancer to the pleura in a necrotic lung scenario [12].

#### 3.1.1. Clinical Manifestations and Diagnosis

At the surgery proposal time, nine (35%) patients already had a previously known UPIPA diagnosis. The age at diagnosis averaged 34.7 years (median 34.5 years, range 17–70 years). The mean time from diagnosis to surgery was 1.4 years (median 0 years, range 0–8 years). The symptom history was available in 24 cases (92%), with a positive history for UPIPA symptoms (i.e., hemoptysis, recurrent respiratory tract infections, and dyspnea) present in 20 (83%) patients. Hemoptysis was the sole onset symptom in nine (38%) cases and was combined with other symptoms (i.e., recurrent respiratory tract infections and dyspnea) in four (17%). Recurrent respiratory tract infections were the isolated first manifestation in five (21%) cases but were associated with other symptoms (i.e., hemoptysis, dyspnea, and productive cough) in six (25%) cases. Only one patient (4%) remained asymptomatic at surgery. Asymptomatic cases involved elderly patients, and the diagnosis was incidental. The precise timing of symptom debut was recorded in 16 (80%) cases at a mean age of 30.5 years (median 24.5 years, range 8–70 years). The mean time from the onset of symptoms to diagnosis was 6.8 years (median 2 years, range 0–38 years), and the mean time from the onset of symptoms to surgery was 8.5 years (median 7 years, range 0–30 years). See Table 1 and Supplementary Table S1 for further details.

Pulmonary hypertension was assessed via echocardiography in 10 cases and with cardiac catheterization in 5. Only two (13%) patients exhibited asymptomatic mildly elevated pulmonary pressures. One of these patients was reevaluated at 6 months from pneumonectomy, revealing lower pulmonary pressure values. The presence of pulmonary hypertension did not affect the postoperative outcomes.

#### 3.1.2. Surgical Details

Pneumonectomy was predominantly conducted via open surgery (only one case via VATS). The approach was commonly a posterolateral thoracotomy, but lateral thoracotomy was also described [14,24]. A bronchial stump coverage was reported only in five articles and was performed by intercostal muscle flap, pericardial flap, or pleural flap. Nine (36%) patients experienced postoperative complications, encompassing thoracotomy wound dehiscence (one, 4%), bronchopleural fistula (after discharge, requiring readmission) treated with reoperation and subsequent thoracostomy (one, 4%), blood clots in the contralateral bronchial tree with respiratory failure requiring bronchoscopy (one, 4%), hemothorax with reoperation (two, 8%), atrial fibrillation (two, 8%), anemia treated with blood transfusions (one, 4%), delirium with rhabdomyolysis, and postoperative ileus (one, 4%). According to the Clavien–Dindo classification, the complications were:

- Three (12%) grade I;
- Two (8%) grade II;
- One (4%) grade IIIa;
- Three (12%) grade IIIb cases.

Complications were more frequent in the urgent/emergent surgery group, with a relative risk of 2.3 (95% CI, 0.7–7.4), though this difference was not statistically significant (*p* = 0.15). Sixteen (64%) patients experienced complications, and early postoperative mortality was null. The hospital length of stay was reported in 16 (64%) cases, with a mean of 8.7 days (median 8.5 days, range 3–16).

#### 3.1.3. Percutaneous Embolization Role

Eleven (44%) patients underwent at least one percutaneous embolization procedure.

- A preoperative embolization was performed to prevent intraoperative bleeding in six (60%) of them. The setting was:
  ○ Lung cancer in one case;
  ○ Hemoptysis in the other five.
- In one additional patient, embolization was employed preoperatively to close a bronchial-coronary fistula.
- In the acute massive hemoptysis setting (seven patients), percutaneous embolization:
  ○ Was attempted in four (57%) cases;
  ○ Was deemed incapable of stopping the bleeding in other two (40%) cases;
  ○ Failed to stop hemoptysis in two (50%) cases;
  ○ Was considered too risky (e.g., medullary ischemia) in one case (25%);
  ○ Was performed preoperatively prior to pneumonectomy in another case (25%).
- In the chronic hemoptysis setting (13 patients), recurrence of hemoptysis after one or more embolizations occurred in five (38%) cases, two of which were early failures. In three cases, this recurrence guided the indication for pneumonectomy.

Two (18%) cases of complications were reported: one patient experienced chest pain in the 24-h post-procedure period, and one patient had a transient ischemic attack.

#### 3.1.4. Intraoperative and Pathological Findings

A prevalent intraoperative finding was the presence of numerous, dense, and vascularized pleural adhesions containing systemic collateral vessels arising from the mediastinum, diaphragm, and thoracic wall. The control may be technically difficult, especially in the diaphragm area [24]. A clear pulmonary artery was not typically found.

The pathological finding of a fibrous cord in the location of the pulmonary artery was reported in only three papers. Lung parenchyma appeared variably damaged: emphysema/bullous dystrophy, fibrosis, bronchiectasis, cystic involution, bronchovascular elements hypoplasia, chronic pleuritis, and intra-alveolar hemorrhage were described.

#### 3.1.5. Follow-Up

Follow-up data were documented in 18 (72%) cases, with additional data obtained by contacting the authors in 1 instance. The follow-up duration was less than 6 months in four (21%) cases, and between 6 and 9 months in four (21%) cases, and it ranged from at least 1 year to a maximum of 3 years in eleven (58%) cases. One patient died of cancer recurrence after one year from surgery [17]. Notably, no surgical complications were reported.

### 3.2. Pneumonectomy in Pediatric Population

In the pediatric UPIPA population, seven pneumonectomies were performed. Patient characteristics are reported in Supplementary Table S2.

The age at surgery ranged from 0 to 9 years. Four patients were under three months old: a few days (one), twenty-three days (one), one month (one), and three months (one). The remaining three patients underwent surgery between 11 months and 9 years: 11 months (one), 7 years (one), and 9 years (one).

Among these cases, five were right UPIPA and two left UPIPA. Two cases presented associated cardiovascular congenital anomalies, one with right UPIPA with a scimitar syndrome (i.e., direct venous drainage of lower lung lobe in inferior vena cava, right lung hypoplasia, heart dextrorotation, and systemic blood supply to right lung) and a patent foramen ovale, and the other with left UPIPA with an interventricular septal defect and right aortic arch. In addition, one patient had a single left pulmonary vein.

All pediatric patients who underwent pneumonectomy were symptomatic. Indications for pneumonectomy included:

- Respiratory and heart failure in a 3-month-old patient after a failed attempt at percutaneous embolization;
- Massive hemoptysis in an 11-month-old patient after a failed attempt of percutaneous embolization and a prior attempt of surgical correction of the PA defect at 7 months;
- Sepsis in two cases (aged 23 days and 1 month);
- Recurrent pneumothorax with cystic lung in a 3-month-old patient;
- Recurrent respiratory tract infections were reported but not clearly defined in a 7-year-old patient;
- Exertional hemoptysis in an 8-year-old patient.

The regimen of pneumonectomy was elective in two (26%) patients and urgent/emergent in five (74%), and, in these cases, pneumonectomy was performed as a life-saving procedure.

#### 3.2.1. Clinical Manifestations and Diagnosis

Similar to adults, common symptoms in pediatric patients included recurrent respiratory tract infections and hemoptysis. However, more severe manifestations, such as heart and respiratory failure, sepsis, and pneumothorax, were observed. In two cases, initial medical treatment was attempted, but the disease eventually progressed. In one of them, an attempt at surgical anastomosis was made but was aborted, given the absence of the distal PA. Only the correction of the associated interventricular septal defect and a lung biopsy were performed. Two months later, the patient required pneumonectomy for hemoptysis. Almost every patient had experienced clinical abnormalities since birth, from failure to thrive to systolic ejection murmur upon auscultation.

In three (60%) patients, pulmonary hypertension was identified. Both echocardiography and cardiac catheterization were performed for diagnosis. After surgery, pulmonary hypertension resolved in all cases.

#### 3.2.2. Surgical Details

Pneumonectomy was exclusively performed via open surgery (100%). In one case, the bronchial stump was covered with a latissimus dorsi muscle flap, phrenemphraxis was performed to elevate the hemidiaphragm, and an inflatable silastic prosthesis was positioned to avoid deformities and mediastinal shift (along with airway collapse due to bronchomalacia) [29]. In another case, a mediastinal tent was created to cover the bronchial stump. No early postoperative complications were reported, and postoperative mortality was null. Interestingly, there was a case of airway collapse due to bronchomalacia after a mediastinal shift in a patient at 2.5 years old after a right pneumonectomy was performed at 1 month of age. Pericardiopexy and aortopexy to the anterior chest wall, along with the positioning of a silastic prosthesis, were performed [33]. Another case experienced a mediastinal shift nine months after surgery and underwent the placement of non-absorbable material in the empty hemithorax, with no further details provided [32].

In three (43%) cases, the hospital length of stay was reported, with a mean duration of 7.6 days and a range of 6–9 days.

#### 3.2.3. Follow-Up

Follow-up data were reported in six (86%) cases. The duration of follow-up ranged from 9 months to 6 years, with a mean of 34.5 months (median 33 months). In one patient (9 years old at pneumonectomy), 11 insufflations of the silastic prosthesis were performed during the 5 years of follow-up, with the last at 14 years old [27]. In another patient (three months old at surgery), a significant mediastinal shift was described at nine months of follow-up, requiring further filling of the empty hemithorax [30]. No long-term mortality was recorded.

## 4. Discussion

UPIPA continues to pose a significant challenge both in diagnosis and management. Although pneumonectomy may be seen as a resolutive treatment, it is generally considered a “last resort” in the literature. Nevertheless, no dedicated study on this matter has been undertaken. The rationale for complete lung resection in UPIPA is grounded in the fact that the affected lung does not contribute to gas exchange and poses potential harm to the body, with an elevated risk of infections and bleeding [2,37]. In the adult population, the decision to pursue surgical treatment depends largely on the patient’s characteristics (i.e., age, fitness, and comorbidity) and the clinical manifestations of the disease.

We found that hemoptysis and bleeding were the most common indications for pneumonectomy in adults, accounting for 57% of cases, with almost half of them attributed to massive hemoptysis. Almost 46% of patients who received pneumonectomy had hemoptysis as the debut symptom, whether isolated (75%) or combined with other symptoms. It is reasonable to think that this group of patients is at higher risk of receiving surgery during life. In older patients (aged > 50), suspected or diagnosed lung cancer was another common surgical indication. Whether UPIPA favors the genesis of lung cancer has not been analyzed yet; however, if UPIPA determines a chronic inflammatory status in the lung, probably mainly due to recurrent infections, it is reasonable to suspect a relationship between the two. Recurrent respiratory tract infections and dyspnea (mainly exertional) may also be indications for surgery. It is reasonable to think that pneumonectomy should be proposed if the quality of life is importantly reduced, given an acceptable fitness level. Finally, imaging, such as chest X-ray and CT scan of the thorax, plays a crucial role in aiding diagnosis and determining the extent of lung damage (e.g., severe emphysema, cystic degeneration, and severe bronchiectasis) to guide surgical decision [37,38].

Percutaneous embolization as the only treatment for massive hemoptysis failed to control symptoms in more than half of the cases in our series. In chronic hemoptysis, recurrence after one or more embolization was seen in more than one-third of patients, and this may eventually lead to surgical indication. In a case series on adults affected by isolated UPIPA, hemoptysis recurred after percutaneous embolization in 83% of cases [2]. In our study, no recurrence of hemoptysis after pneumonectomy was found.

In our study, pneumonectomy was mainly performed in young adult patients, with a mean age of 39 years, and around half (44%) received an urgent/emergent procedure. Nearly 36% of patients experienced an early postoperative complication, of which 12% were classified as Clavien–Dindo grade IIIb. Atrial fibrillation occurred in 8% of patients, always aged >40 years old. As expected, pneumonectomy was confirmed to have a significant risk of morbidity. However, it seems cautious to affirm that usual patients (i.e., older adults affected by lung cancer, undergoing the procedure in an elective fashion) are different from those analyzed in this study; thus, a reliable comparison cannot be made [39]. It may be rational to suppose that a younger patient may better bear a pneumonectomy. So, given its resolutive effect on UPIPA, it may be offered to these patients, depending on their fitness and symptom severity. On the contrary, older asymptomatic patients may benefit less from pneumonectomy. From a postoperative standpoint, as demonstrated by the case of Rousou et al., it seems cautious to suggest the positioning of a chest tube at the end of the surgery, which allows for the monitoring of eventual bleeding [10].

Preoperative embolization was performed in 24% of adult cases undergoing pneumonectomy. This procedure may help reduce postoperative bleeding in a high-risk population, such as patients with hemoptysis as the primary symptom. However, the effectiveness of this intervention remains challenging to evaluate without a comparison group.

If pneumonectomy may appear as the only resolutive surgical solution for adults affected by UPIPA, in the pediatric population, the first attempt should be to reobtain normal arterial perfusion. In fact, anastomosis between the main pulmonary artery and distal pulmonary artery has been described with satisfactory outcomes [40,41,42]. Moreover, pneumonectomy in the child brings important consequences related to subsequent growth. So, it should be proposed only when facing life-threatening complications [28,33] and when an arterial anastomosis is not feasible [31]. It is reasonable to eventually consider pneumonectomy after growth completion if the clinical manifestations are not urgent or disabling. However, it should be noted that the pediatric cohort included only seven patients, thus limiting the quality of evidence.

A possible post-pneumonectomy late complication in children is mediastinal shift with airway compression due to tracheo- or broncho-malacia. Among six children, this complication appeared in 17% of cases. The positioning of an inflatable prosthesis aimed to mitigate mediastinal shift, bronchomalacia, and deformities (i.e., chest wall deformities and scoliosis). During follow-up, the patient would require several adjustments. In the study of Nichol et al., the last inflation was performed at 14 years old, after 11 inflations during a 5-year follow-up period [29]. Another possible technique to relieve airway compression is the suspension of the pericardium and aorta to the thoracic wall (pericardiopexy and aortopexy). However, this is associated with a high rate of recurrence [33], which is the reason why it is usually combined with other techniques.

To the best of our knowledge, this is the first review concerning pneumonectomy in UPIPA patients. While it provides insights into this issue, the limitations should be acknowledged. Even if it is a systematic review, the rarity of UPIPA and the infrequent use of pneumonectomy as a treatment limit the number of cases available for analysis. For this reason, we tried to extend the inclusion criteria as much as possible to maximize the number of analyzed cases. While it is not possible to cover all the additional cases that were not found in the literature, it is reasonable to think they exist. In addition, the quality of the data is limited due to the nature of the included studies: case reports and case series. Finally, we performed an analysis on UPIPA patients that received pneumonectomy, not on all UPIPA cases, thus limiting a comprehensive understanding of this disease and its optimal management.

## 5. Conclusions

The management of UPIPA presents a complex clinical challenge. Pneumonectomy achieves complete resolution of UPIPA symptoms. In the adult population, its primary indication is hemoptysis, with procedures conducted in both elective and urgent/emergent settings. Despite a mortality rate of zero, a notable proportion of patients may experience postoperative complications. In pediatric cases, the clinical presentation varies more extensively, and pneumonectomy is typically reserved for life-threatening situations, emphasizing the need for careful patient selection.

## Figures and Tables

**Table 1:** Adult UPIPA patients’ characteristics. Debut symptoms: first-reported symptoms of the disease (from past medical history or first presentation). * Two articles are not included, given the absence of such information. ** Calculated from a total of 26 patients.

Adult Patients (N = 26)	Value
Mean age at surgery (years)	39.2
Gender	
Male	15 (58%)
Female	11 (42%)
UPIPA side	
Right	15 (58%)
Left	11 (42%)
UPIPA type	
Isolated	17 (65%)
Combined	9 (35%)
of which:	
right aortic arch	8 (89%)
aberrant subclavian artery with Kommerell’s diverticulum	1 (11%)
Debut symptom (n = 24) *	
Hemoptysis only	9 (38%)
Recurrent respiratory infections only	5 (21%)
Mix of hemoptysis, recurrent respiratory infections, dyspnea, and chest pain	9 (38%)
Asymptomatic	3 (12% **)
Mean time symptoms onset—diagnosis (years)	6.8
Mean time symptoms onset—pneumonectomy (years)	8.5
Indication to pneumonectomy	
Massive acute hemoptysis	7 (27%)
Suspected or diagnosed tumor	6 (23%)
New-onset of acute hemoptysis	4 (15%)
Persistent/recurrent hemoptysis	4 (15%)
Mix of hemoptysis, recurrent respiratory tract infections, and dyspnea	3 (12%)
Recurrent respiratory infections in a setting of a damaged lung	1 (4%)
Myocardial ischemia for coronary steal	1 (4%)
Pneumonectomy timing	
Elective	14 (54%)
Urgent/emergent	12 (46%)
Postoperative complications (n = 25)	9 (36%)

## Data Availability

The data presented in this study are available on request from the corresponding author.

## Supplementary Materials

The following supporting information can be downloaded at https://www.mdpi.com/article/10.3390/life13122328/s1. Supplementary Figure S1. Chest-X-ray of the patient at diagnosis. Supplementary Figure S2. CT scan of the thorax of the patient at diagnosis. Supplementary Video S1. 3D reconstruction of the CT scan of the great vessels and the heart. Supplementary Video S2. Angiography sequences and embolization of major systemic collaterals (phrenic, intercostal, bronchial, and inferior thyroid arteries). Supplementary Video S3. Intraoperative view of the intercostal muscle flap on the right bronchial stump. Supplementary Table S1. Adult UPIPA patients’ characteristics. Supplementary Table S2. Pediatric UPIPA patients’ characteristics. Supplementary Text S1. Case report.
