# Supplementary material for: Pneumonectomy for Unilateral Proximal Interruption of Pulmonary Artery: A Case Series from the Literature
Source: Life (Basel). 2023 Dec 12;13(12):2328. doi: 10.3390/life13122328 (PMC10744847; doi:10.3390/life13122328)
Supplement: Supplementary file 1 [file life-13-02328-s001.zip › Supplementary Tables and Figures.pdf]

Supplementary materials

Supplementary Table S1. Adult UPIPA patients' characteristics. y=years, m=months, d=days; Y=YES, N=NO, U=unknown. E=elective, Ur/Em=urgent/emergent, F-UP=follow-up. NA=not applicable. Debut symptoms: first reported symptoms of the disease (from past medical history or first presentation). Evolution of symptoms: reported symptoms after the appearance of the debut symptoms (if the case report describes the first presentation, NA is here written).

Authors	Year	Age at surgery	Gender	UPIPA side	Associated anomalies	Debut symptoms (diagnosis was made? Y/N)	Evolution of symptoms (diagnosis was now made? Y/N/U)	Indication to pneumonectomy (diagnosis needed? Y/N)	Elective (E) or Emergent/Urgent (U)	Onset to diagnosis time (years)	Diagnosis to Surgery time	Complications	F-UP
Smith NE et al. [5]	2018	35	M	Right	None	Recurrent respiratory tract infections (N)	Recurrent respiratory tract infections (Y)	New-onset acute hemoptysis (Y)	E	20	0	Not known	2 months
Kang MK et al. [6]	2019	21	M	Right	None	Persistent/recurrent hemoptysis (N)	Persistent/recurrent hemoptysis (N)	Massive acute hemoptysis (Y)	U	5	0	Thoracotomy wound dehiscence	2 months
De Dominicis F et al. [7]	2011	44	F	right	None	Recurrent respiratory tract infections (U)	Recurrent respiratory tract infections (U)	Myocardial ischemia for coronary steal (N)	U	Not known (months-years)	Not known (months-years)	Atrial fibrillation	6 months
Wang J et al. [8]	2020	56	M	Left	Right aortic arch	Chest pain (Y)	NA	Suspected or diagnosed tumour (Y)	E	0	0	No	2 years
Britton J et al. [9]	2011	21	F	Right	None	Persistent/recurrent hemoptysis (N)	Persistent/recurrent hemoptysis (Y)	Massive acute hemoptysis (N)	U	9	4	No	4 months
Rousou AJ et al. [10]	2009	34	M	Left	Aberrant subclavian artery from Kommerell's diverticulum	New-onset acute hemoptysis (Y)	NA	New-onset acute hemoptysis (Y)	E	0	0	Reoperation for hemothorax	8 months
Makdisi G et al. [11]	2015	50	F	Right	None	Exertional dyspnea, persistent/recurrent hemoptysis (N)	Exertional dyspnea, persistent/recurrent hemoptysis (Y)	Suspected or diagnosed tumour (Y)	E	30	0	Atrial fibrillation	1 year
Agzarian J et al. [12]	2019	55	F	Left	None	Recurrent respiratory tract infections (Y)	Productive cough, recurrent respiratory tract infections (N)	Suspected or diagnosed tumour (N)	U	0	8	NA	Not known
Griffin N et al. [13]	2007	33	M	Right	None	Persistent/recurrent hemoptysis, recurrent respiratory tract infections (N)	Exertional dyspnea, persistent/recurrent hemoptysis, recurrent respiratory tract infections (Y)	Exertional dyspnea, persistent/recurrent hemoptysis, recurrent respiratory tract infections (N)	E	Not known (months-years)	2	No	Some months
Griffin N et al. [13]	2007	21	F	Right	None	Persistent/recurrent hemoptysis (N)	Exertional dyspnea, persistent/recurrent hemoptysis (Y)	Suspected or diagnosed tumour (Y)	E	Not known (months-years)	0	No	Not known
Al Jabbar	2016	52	F	Left	Right aorti	Exertional dyspnea,	Exertional dyspnea,	Exertional dyspnea,	E	Not known	Not know	No	6 months

i O et al. [14]					c arch	recurrent respiratory tract infections (N)	recurrent respiratory tract infections, productive cough, persistent/recurrent hemoptysis (Y)	recurrent respiratory tract infections, persistent/recurrent hemoptysis (N)		(months-years)	n (months-years)		
Betigeri VM et al. [15]	2013	35	M	Right	None	Recurrent respiratory infections, exertional dyspnea, productive cough (N)	Recurrent respiratory infections, exertional dyspnea, productive cough (Y)	Recurrent respiratory infections in damaged lung (Y)	E	8	0	No	2 years
Redmond KC et al. [16]	2009	70	M	Left	Right aortic arch	Exertional dyspnea and productive cough (Y)	NA	Suspected or diagnosed tumour (Y)	E	0	0	No	18 months
Kononets PV et al. [17]	2022	59	F	Left	Right aortic arch	None (Y) with lung mass at chest X-ray	NA	Suspected or diagnosed tumour (Y)	E	0	0	No	11 months
Maeda S et al. [18]	2001	19	F	Right	None	Persistent/recurrent hemoptysis (N)	Persistent/recurrent hemoptysis (Y)	Persistent/recurrent hemoptysis (Y)	E	8	0	Not known	Not known
Bekoe S et al. [19]	1993	26	F	Right	None	Exertional dyspnea, chest pain (N)	Exertional dyspnea, chest pain, new-onset acute hemoptysis (Y)	New-onset acute hemoptysis (Y)	U	1	0	No	3 years
Jiang Y et al. [20]	2006	38	M	Right	None	Not known	Not known	New-onset acute hemoptysis (Y)	E	Not known (months-years)	0	No	Not known
Ohtsuka T et al. [21]	2006	48	M	Right	None	Persistent/recurrent hemoptysis (U)	Persistent/recurrent hemoptysis (U)	Persistent/recurrent hemoptysis (Y)	E	3	0	No	6 months
de Mello Junior WT et al. [22]	2008	30	M	Right	None	Not known	Not known	Massive acute hemoptysis (Y)	U	Not known	0	No	Not known
Thomas P et al. [23]	2001	35	F	Left	Right descending aorta	Recurrent respiratory tract infections (N)	Recurrent respiratory tract infections, persistent/recurrent hemoptysis, exertional dyspnea (N)	Massive acute hemoptysis (Y)	U	25	0	Desaturation due to blood clots in the contralateral bronchial tree	18 months
Farghly E et al. [24]	2002	35	M	Left	Right aortic arch	Exertional dyspnea, recurrent respiratory tract infections (N)	Exertional dyspnea, persistent/recurrent hemoptysis, recurrent respiratory tract infections (N)	Persistent/recurrent hemoptysis (Y)	E	Not known (months-years)	0	Reoperation for hemothorax	18 months
Sanna S et al. [25]	2012	31	F	Rght	None	Exertional dyspnea, recurrent respiratory tract infections (N)	Exertional dyspnea, recurrent respiratory tract infections (Y)	Massive acute hemoptysis (N)	U	Not known (months-years)	4	Bronchopleural fistula (readmission)	1 year
Ciura VA et al. [26]	2008	30	M	Left	Right aortic arch	Persistent/recurrent hemoptysis (Y)	Persistent/recurrent hemoptysis (N)	Massive acute hemoptysis (N)	U	0	6	Not known	Not known
Maini A et al. [27]	2023	55	M	Left	Right aortic arch, singles left	New-onset acute hemoptysis (Y)	Persistent/recurrent hemoptysis (N)	Persistent/recurrent hemoptysis (N)	U	Not known (months - years)	Not known (several years)	Delirium, rhabdomyolysis, postope	1 year

					pulm onar y vein							rative ileus	
Roma n J et al. [35]	199 5	54	M	Left	Right aorti c arch	Recurrent respiratory tract infections (N)	Recurrent respiratory tract infections (N)	Suspected or diagnosed tumour (Y)	U	Not known (months -years)	0	No	Not known
Presen t study	202 3	31	M	Right	None	Exertional dyspnea, recurrent respiratory tract infections, persistent/re current hemoptysis (Y)	Exertional dyspnea, recurrent respiratory tract infections, persistent/rec urrent hemoptysis (N)	Massive acute hemoptysis (N)	U	0	8	Anemia	1 year

Supplementary Table S2. Pediatric UPIPA patients' characteristics. y=years, m=months, d=days; Y=YES, N=NO, U=unknown. E=elective, Ur/Em=urgent/emergent, F-UP=follow-up. NA=not applicable. Debut symptoms: first reported symptoms of the disease (from past medical history or first presentation). Evolution of symptoms: reported symptoms after the appearance of the debut symptoms (if the case report describes the first presentation, NA is here written).

Author s	Yea r	Age at surge ry (y, m or d)	Gend er	UPIP A side	Associated anomalies	Debut symptoms (diagnosis was made? Y/N)	Evolution of symptoms (diagnosis was now made? Y/N/U)	Indication to pneumonectom y (diagnosis needed? Y/N)	E or Ur/E m	Onset to diagno sis time (y, m or d)	Diagno sis to Surgery time (y, m, or d)	Complicati ons	F-UP
Canver CC et al. [28]	199 1	23 d	F	Righ t	None	Respirator y failure, sepsis (Y)	NA	Respiratory failure, sepsis (Y)	U	0	0	No	1 y
Nichol PF et al. [29]	200 4	9 y	F	Righ t	None	None (N) with chest X-ray abnormalit ies	Persistent/recur rent hemoptysis (Y)	Persistent/recur rent hemoptysis (Y)	E	1 y	0	No	6 y
Beitzke A et al. [30]	199 2	2 m	F	Righ t	Scimitar syndrome, patent FO e patent DA (shunt left to right)	Failure to thrive (Y)	Heart failure, respiratory failure (N)	Heart failure, respiratory failure (N)	U	0	2 m	No	3 y
Imanak a K et al. [31]	199 8	11 m	M	Left	Interventric ular sept defect, right aortic arch, patent DA, single left	Dyspnea, systolic murmur (Y)	Recurrent respiratory tract infections (N)	Recurrent respiratory tract infections (N)	U	0	9 m	No	4 y

					pulmonary vein								
George A et al. [32]	2022	3 m	M	Right	None	Respiratory failure, recurrent PNX (Y)	Recurrent PNX (N)	Recurrent PNX (N)	U	3 m	0	Late mediastinal shift (requiring hemitorax volume filling)	9 m
Newman R et al. [33]	1994	1 m	F	Right	None	Sepsis (Y)	NA	Sepsis (Y)	U	0	1 m	No	10 m
Anand SH et al. [34]	2015	7 y	M	Left	None	Recurrent respiratory infections (N)	Recurrent respiratory infections (Y)	Not known (Y)	E	Not konwn	0	Not known	Not konw

Figures and videos legends

Supplementary Figure S1. Chest-X-ray of the patient at diagnosis. The right lung appears hypoplastic compared to the left one and has prominent bronchovascular markings. The left lung appears hyperlucent. The trachea is shifted towards the diseased lung.

Supplementary Figure S2. CT scan of the thorax of the patient at diagnosis. A=sagittal view of the hilum of the right UPIPA lung, B= sagittal view of the hilum of the left normal lung, C and E=coronal view, D=transverse view. A clear right pulmonary artery is not recognizable. A tangle of systemic collaterals can be clearly seen in the mediastinum (white arrow in C and yellow triangle in D), perfusing the right UPIPA lung. An ectatic phrenic artery can be appreciated over the diaphragm (yellow triangle in C).

Supplementary Video S1. 3D reconstruction of the CT scan of the great vessels and the heart.

Supplementary Video S2. Angiography sequences and embolization of major systemic collaterals (phrenic, intercostal, bronchial, and inferior thyroid arteries).

Supplementary Video S3. Intraoperative view of the intercostal muscle flap on the right bronchial stump.
