# Supplementary material for: Pneumonectomy for Unilateral Proximal Interruption of Pulmonary Artery: A Case Series from the Literature
Source: Life (Basel). 2023 Dec 12;13(12):2328. doi: 10.3390/life13122328 (PMC10744847; doi:10.3390/life13122328)
Supplement: Supplementary file 1 [file life-13-02328-s001.zip › Text S1. Case report.pdf]

Case report (Supplementary Text S1)

We present the case of a 30-year-old male patient who experienced a pneumonia episode at the age of eight, necessitating hospitalization. Subsequently, he started smoking, and at the age of 22, he exhibited significant hemoptysis associated to exertional dyspnea. Diagnostic investigations were started, including chest-X-ray, bronchoscopy (yielding negative results), and chest CT scan, and led to the diagnosis of a right isolated unilateral proximal interruption of pulmonary artery (UPIPA). This anomaly was characterized by an hypoplastic affected lung and a compensatory systemic arterial circle involving intercostal, bronchial, inferior thyroid, and phrenic ectatic arteries (see Supplementary Figure S1, Supplementary Figure S2 and Supplementary Video S1). Considering the patient's stability, a conservative treatment approach was initiated with tranexamic acid and antibiotic therapy, followed by referral to our institution. Echocardiography showed an increase in left ventricular thickness, and an ejection fraction of 51% without pulmonary hypertension, and lung perfusion scintigraphy indicated no right lung perfusion. Respiratory function test revealed an obstructive deficit, and the cardiopulmonary exercise test identified a mild deficit. Given the clinical status (e.g., age, symptoms, fitness) consensus was reached on the indication for open right pneumonectomy. Initially, the patient declined. Several years passed by and he experienced multiple hemoptysis episodes, persistent exertional dyspnea, one hospitalization, and three additional CT scans. At the age of 30, the patient agreed to surgery and underwent anesthesiologic evaluation. During the evaluation, a massive hemoptysis occurred, necessitating urgent hospitalization. Before proceeding with pneumonectomy, percutaneous embolization was performed, effectively using coils and glue to close all bleeding and ectatic vessels (see also Supplementary Video S2). The following day, an open right pneumonectomy was performed via posterolateral thoracotomy. In the operating room, ECMO was available with peripheral access obtained. Many tenacious pleuropulmonary adhesions were encountered and they were particularly vascularized. The most complex dissection occurred at the mediastinal side, which included several ectatic bronchial arteries, and completely absent pulmonary artery. An intercostal muscle flap was positioned on the bronchial stump (see also Supplementary Video S3). The operative time was 253 minutes. The patient received 22 units of red blood cells during the operation. The postoperative course was characterized only by anemia, for which the

patient received two additional red blood cells units. The chest tube was removed on postoperative day six and the patient was discharged on postoperative day nine. Pathological analysis of the specimen revealed chronic pleuritis with pleural thickening and intralveolar hemorrhage. After more than one year from surgery, the patient remains well with no complaints or symptoms recurrence (i.e., hemoptysis and exertional dyspnea). He expressed that he would have consented to surgery earlier if he could go back in time.

The patient provided informed consent both for the surgery and the scientific use of clinical data.
