# Supplementary figures and images for: Pneumonectomy for Unilateral Proximal Interruption of Pulmonary Artery: A Case Series from the Literature
Source: Life (Basel). 2023 Dec 12;13(12):2328. doi: 10.3390/life13122328 (PMC10744847; doi:10.3390/life13122328)

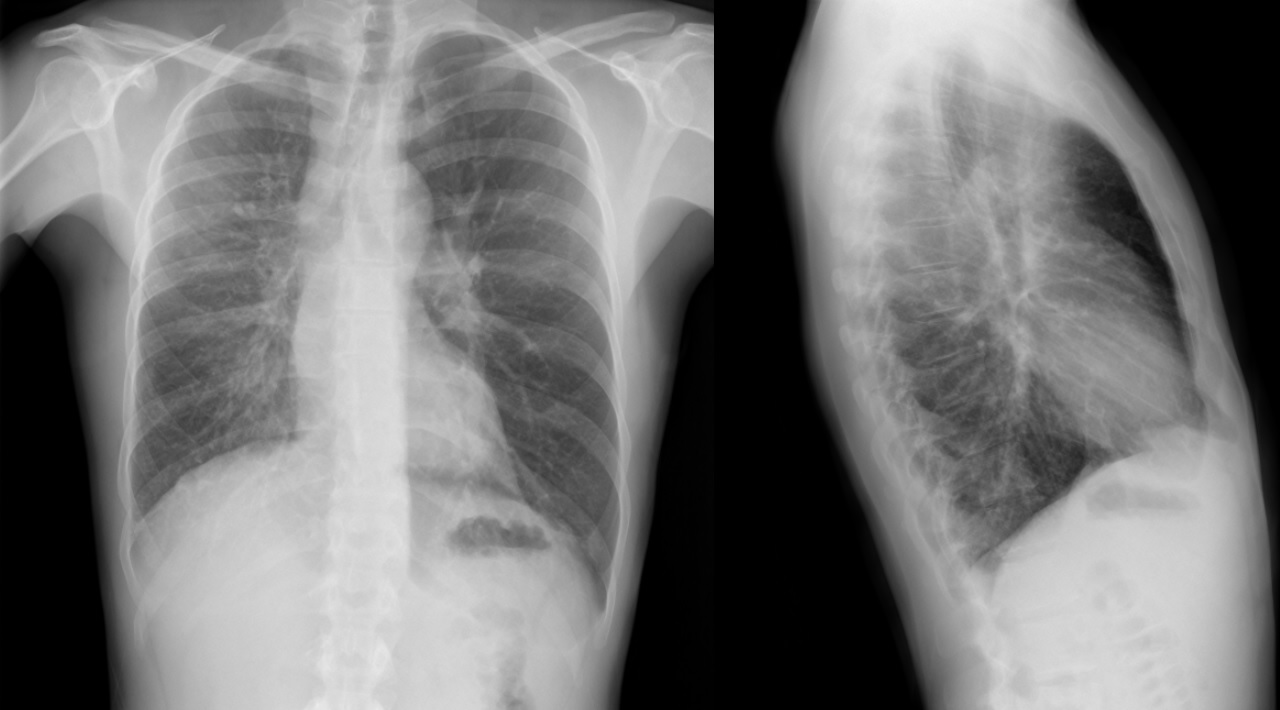

Supplement: Supplementary file 1 [file life-13-02328-s001.zip › Supplementary Figure S1.jpg]

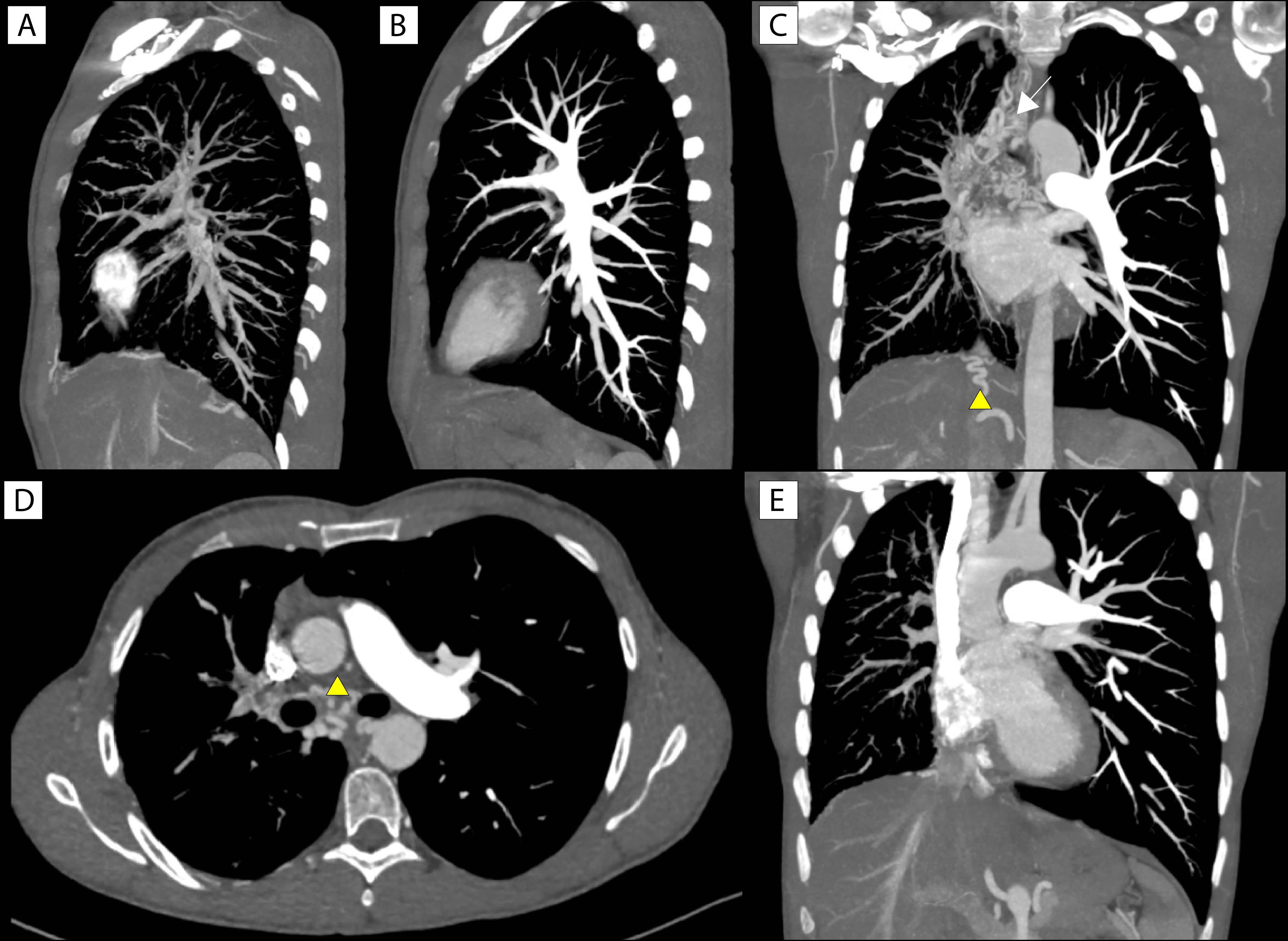

Supplement: Supplementary file 1 [file life-13-02328-s001.zip › Supplementary Figure S2.jpg]
